# Draft genome sequence of a promising bioremediation agent *Gordonia paraffinivorans* IEGM 735

**DOI:** 10.1128/mra.01059-25

**Published:** 2026-02-09

**Authors:** Anastasia Golysheva, Anastasiia Krivoruchko, Irena Ivshina

**Affiliations:** 1 Perm Federal Research Centre 307499, Perm, Russia; 2 Perm State University 64949, Perm, Russia; University of Southern California, Los Angeles, California, USA

**Keywords:** *Gordonia paraffinivorans*, bioremediation, hydrocarbons, heavy metals

## Abstract

A high-quality draft genome sequence of actinomycete *Gordonia paraffinivorans* IEGM 735 isolated from wastewater was obtained using Illumina sequencing. This genome sequence will be useful for elucidating the genetic mechanisms of xenobiotic biodegradation and heavy metal resistance within a rare, underexplored, and biotechnologically promising species *G. paraffinivorans*.

## ANNOUNCEMENT

Non-pathogenic actinomycetes are effective bioremediation agents (1–5). *Gordonia paraffinivorans* is a rare and underexplored species, tolerating heavy metals and degrading hydrocarbons, polyesters, and rubber (6–9). *G. paraffinivorans* IEGM 735 was isolated from foam in a wastewater aeration plant in Irvine, Scotland, UK. A 1 mL of undiluted sample collected aseptically was spread on a plate with mineral agar K (http://www.iegmcol.ru/medium/med08.html) and a sterile filter paper saturated with 0.2 mL of sterile *n-*hexadecane (>97% purity) under the lid. Plates (three replicates) were incubated at 28°C for 1 week. To obtain a pure culture, the third generation was used, which was obtained by streaking target colonies on a fresh medium. The strain was preliminarily identified as *Gordonia terrae* (NCBI:txid2055) using phenotypic tests and 16S rRNA gene analysis. A genetic analyzer 3500 (Applied Biosystems, Waltham, MA, USA) and primers 27F/1492R (https://kb.ezbiocloud.net/home/science-blogs/identify/16s-rrna) were used to sequence the 16S rRNA gene after the isolation of genomic DNA with a kit ExtractDNA Blood&Cells (Evrogen, Moscow, Russia). The strain was deposited in the Regional Specialised Collection of Alkanotrophic Microorganisms (acronym IEGM, WDCM #768, http://www.iegmcol.ru/strains/gordonia/paraffinivorans/g_paraffinivorans735.html).

The cells were recovered from a lyophilized culture stored for 21 years and grown in Luria-Bertani (LB) broth at 28°C and 160 rpm for 28 h. DNA was isolated from the pure culture using the Quick-DNA Fungal/Bacterial Microprep Kit (Zymo Research, Irvine, CA, USA) following the manufacturer’s protocol and quantified using a Qubit 4 Fluorometer (Thermo Fisher Scientific, Waltham, MA, USA). The quality was assessed by 1% agarose gel electrophoresis. The DNA concentration was 139 ng/μL (total 12,232 ng). A total of 100 ng DNA was used for sequencing. A paired-end library (2 × 100 nucleotides [nt]) was produced using Illumina DNA Prep and sequenced on an Illumina NovaSeq 6000 instrument (Illumina, Inc., San Diego, CA, USA). The library’s quality was estimated using a fluorescence analysis and a 2100 Bioanalyzer (Agilent Technologies, Santa Clara, CA, USA). A total of 22,370,745 reads were obtained. Demultiplexing was performed using Illumina bcl2fastq 2.20. Adapters were trimmed using Skewer 0.2.2 (10). The quality of FASTQ files was analyzed using FastQC 0.11.5-cegat (11). The data were assembled using SPAdes 3.14.1 (12). The assembly consisted of 65 contigs with a total length of 4,452,968 bp, an N50 value of 277,909 bp, a guanine-cytosine (GC) content of 69%, and a coverage of 506×, indicating a high-quality sequence (13). Average nucleotide identity (ANI) and digital DNA/DNA hybridization (dDDH) values were calculated using ANI Calculator (https://www.ezbiocloud.net/tools/ani) (14), Genome-to-Genome Distance Calculator 3.0 (https://ggdc.dsmz.de/ggdc.php#) (15), and TYGS (https://tygs.dsmz.de/) (15, 16). As ANI was 99.05% and dDDH was 88.00%–92.30%, IEGM 735 was reclassified as *G. paraffinivorans* (NCBI:txid175628). The annotation was performed using PGAP 6.8 (17). The default parameters were used for all software applications unless otherwise specified.

A total of 4,036 CDS, 3,940 CDS with protein, and 54 RNAs were found. Genes coded for 16 metal/cation transporters, 133 ABC transporters, 10 cation antiporters, 7 metalloregulators, 16 heavy metal resistance proteins, 9 universal stress proteins, 10 cytochrome c oxidases, and 10 cytochromes P450 were identified.

## Data Availability

This Whole-Genome Shotgun project has been deposited in DDBJ/ENA/GenBank under the accession numbers JAJNDG010000001–JAJNDG010000065 at https://www.ncbi.nlm.nih.gov/Traces/wgs/JAJNDG01?display=contigs. The version described in this paper is the first version available as assembly GCF_021026155.1. Raw sequence reads were deposited in the Sequence Read Archive with accession number SRR17030447 under BioSample accession number SAMN23420019 and BioProject accession number PRJNA783162. The partial 16S rRNA gene sequence is deposited under the accession number MG912567.
